# Triage test for all-oral drug-resistant tuberculosis (DR-TB) regimen: a phase IV study to assess effectiveness, feasibility, acceptability and cost-effectiveness of the Xpert MTB/XDR assay for rapid triage and treatment of DR-TB

**DOI:** 10.1136/bmjopen-2024-084722

**Published:** 2024-11-27

**Authors:** Kogieleum Naidoo, Anushka Naidoo, Alash'le G Abimiku, Everdina W Tiemersma, Agnes Gebhard, Sabine M Hermans, Derek J Sloan, Morten Ruhwald, Sophia B Georghiou, Evaezi Okpokoro, Aderonke Agbaje, Kalkidan Yae, Getachew Tollera, Shewki Moga, Hannelise Feyt, Takondwa Kachoka, Marothi P Letsoalo, Andrea M Cabibbe, Rubeshan Perumal, Letitia Shunmugam, Daniela M Cirillo, Salah Foraida, Wilber Sabiiti, Nyanda Elias Ntinginya, Bariki Mtafya, Ahmed Bedru, Stephen H Gillespie, on behalf of the TRiAD Study Consortium

**Affiliations:** 1 Centre for the Aids Programme of Research in South Africa, Durban, KwaZulu-Natal, South Africa; 2 SAMRC-CAPRISA HIV-TB Pathogenesis and Treatment Research Unit, Doris Duke Medical Research Institute, University of KwaZulu-Natal, Durban, KwaZulu-Natal, South Africa; 3 International Research Center of Excellence, Institute of Human Virology Nigeria, Abuja, Nigeria; 4 University of Maryland School of Medicine, Baltimore, Maryland, USA; 5 Koninklijke Nederlandse Centrale Vereniging tot Bestrijding der Tuberculose, The Hague, The Netherlands; 6 Amsterdam UMC location University of Amsterdam, Department of Global Health, Amsterdam Institute for Global Health and Development, Amsterdam, The Netherlands; 7 University of St Andrews, St Andrews, UK; 8 Foundation for Innovative New Diagnostics, Geneva, Switzerland; 9 Koninklijke Nederlandse Centrale Vereniging tot Bestrijding der Tuberculose, Addis Ababa, Ethiopia; 10 Ethiopian Public Health Institute, Addis Ababa, Ethiopia; 11 Clinical HIV Research Unit (CHRU), Jose Pearson TB Hospital, Wits Health Consortium Pty Ltd, Port Elizabeth, South Africa; 12 Emerging bacterial pathogens, Ospedale San Raffaele, Milano, Italy; 13 Global Alliance for TB Drug Development, New York, New York, USA; 14 National Institute for Medical Research, Dar es Salaam, United Republic of Tanzania

**Keywords:** Molecular diagnostics, Tuberculosis, Observational Study, Public health

## Abstract

**Introduction:**

The TriAD study will assess the Xpert MTB/XDR (Xpert XDR; Cepheid) assay to detect tuberculosis (TB) drug resistance in sputum testing positive for TB to rapidly triage and treat patients with a short all-oral treatment regimen.

**Methods and analysis:**

In this study, approximately 4800 Xpert MTB/RIF or Ultra MTB-positive patients (irrespective of rifampicin (RIF) resistance (RR) status) from several clinical sites across South Africa, Nigeria and Ethiopia will be enrolled over 18–24 months and followed-up for approximately 6 months post-TB treatment completion. Participants will be enrolled into one of two cohorts based on Xpert MTB/RIF and Xpert XDR results: *Mycobacterium tuberculosis* (*M.tb*) positive participants with RR in Cohort 1 (n=880) and *M.tb* positive RIF susceptible TB patients with isoniazid mono-resistance irrespective of presence of resistance to fluoroquinolones, second-line injectable drugs or ethionamide in Cohort 2 (n=400). Cohort 1 will be compared with historical cohorts from each implementing sites. The primary study outcomes include time to initiation of an appropriate treatment regimen by resistance profile and the proportion of patients with favourable treatment outcomes compared with historical cohorts from each of the implementing sites. Secondary outcomes include feasibility, acceptability and cost-effectiveness of this approach to inform policies and guidelines for programmatic implementation of this triage and treat model for drug-resistant tuberculosis management. Utility of the tuberculosis molecular bacterial load assay (TB-MBLA) for real-time treatment response assessment will also be evaluated.

**Ethics and dissemination:**

The University of KwaZulu-Natal Biomedical Research Ethics Committee (BREC) and local research committees have provided ethical review and approval (BREC/00002654/2021, HREC 210805, NHREC/01/01/2007 and EPHI-IRB-459–2022). The South African Health Products Regulatory Authority (SAHPRA) have granted regulatory approval for the TRiAD Study (SAHPRA MD20211001). Trial results will be disseminated through conference presentations, peer-reviewed publications and the clinical trial registry.

**Trial registration number:**

Clinicaltrials.gov; Trial registration number: NCT05175794; South African National Clinical Trials Register (SANCTR DOH-27-012022-4720)

**STRENGTHS AND LIMITATIONS OF THIS STUDY**

- The study will be the first to demonstrate the ability of the Xpert XDR to reduce time to appropriate treatment start.
- The study includes novel assays such as the tuberculosis molecular bacterial load assay (TB-MBLA), which will be used for the first time to monitor treatment response in drug-resistant tuberculosis (DR-TB) patients.
- The study evaluates the introduction of the 2021 WHO DR-TB guideline in various geographical settings.
- Additionally, this study will be able to assess the effectiveness of rapid diagnosis of fluoroquinolone resistance using Xpert XDR on rapid initiation of bedaquiline-pretomanid-linezolid-moxifloxacin (BPaLM) regimen, for individuals with pre-extensively drug-resistant TB, where early fluoroquinolone resistance detection provides clinical guidance for the regimen to be administered without moxifloxacin (BPaL).
- The study does not include patients with severe allergy to any of the BPaL/BPaLM component drugs or individuals with specific forms of severe TB such as TB meningitis, central nervous system TB or TB osteomyelitis and pregnant or breastfeeding women; hence, study findings are not generalised to these populations.

## Introduction

 Tuberculosis (TB), caused by infection with *Mycobacterium tuberculosis* (*M.tb*), remains one of the deadliest infectious diseases in the world. The burden of multidrug-resistant TB (MDR-TB), characterised by resistance to at least rifampicin (RIF) and isoniazid (INH), has increased by more than 20% per annum, from 2009 to 2017.^1^ Globally in 2021, the prevalence of RIF resistance (RR) and INH resistance, both independently and combined, was 3.6% and 10.7% among new TB patients and 18% and 27.2% among previously treated TB patients.^2^ In 2021, drug-resistant TB (DR-TB) increased by 3%, with 450 000 new cases of RIF-resistant TB (RR-TB) notified, with approximately 25% of the global DR-TB burden in Sub-Saharan Africa.^3^ There is a crucial need for new, effective diagnostic and therapeutic approaches with improved accuracy to replace time-consuming laborious phenotypic methods for timely initiation of treatment and treatment response monitoring in the realm of DR-TB.^4^

Globally, DR-TB treatment success rates are approximately 60%, highlighting the healthcare systems failure to effectively control the epidemic with the current diagnostic and therapeutic strategies.^5^ The treatment of DR-TB is burdensome for patients, involving complex multidrug regimen administered over lengthy treatment periods. These regimens often include drugs that provoke severe toxicities and drug-drug interactions. In recent years, there has been a shift in DR-TB guidelines towards all-oral therapy with the emergence of shorter bedaquiline (BDQ)-based treatment, replacing second-line injectable agents. In December 2022, the WHO guidelines recommended the 6 months BDQ, pretomanid (PMD), linezolid (LZD) and moxifloxacin (MFX) (bedaquiline-pretomanid-linezolid-moxifloxacin (BPaLM)) regimen for patients with MDR-TB/RR-TB or pre-extensively drug-resistant TB (pre-XDR-TB). In situations where resistance to fluoroquinolones (FQs) is confirmed, BPaL treatment without the inclusion of MFX was recommended.^6^

The current front-line molecular diagnostic assay for drug resistance is Cepheid Xpert MTB/RIF or Xpert MTB/RIF Ultra, detecting the presence of *M.tb* while simultaneously identifying mutations associated with RR. Though the test rapidly identifies patients who are eligible for MDR-TB treatment, the test is unfortunately limited to detecting RIF resistance only.^7 8^ Samples showing RIF resistance undergo reflex testing with the Hain Line Probe Assay (LPA; GenoType MTBDRsl), for detection of resistance to second-line TB drugs.^9^ Drug susceptibility testing (DST) for all other drugs in the DR-TB regimen is performed using phenotypic testing methods.^10^ Despite the rapid diagnosis provided by Xpert MTB/RIF or Xpert MTB/RIF Ultra, all follow-on testing is conducted at a central laboratory, which increases the turnaround times for providing a comprehensive resistance profile.

Furthermore, these DST methods have several limitations. While LPA testing can be conducted rapidly on sputum samples, it necessitates specialised laboratory facilities, has longer turnaround time to results, requires skilled interpretation of the test strip and is associated with a high indeterminate rate (~30%) in smear-negative patients.^11^ Phenotypic DST techniques, on the other hand, typically require 4 to 6 weeks to yield results.^12^

In the management of DR-TB, patients are initially started on empiric treatment following the detection of RR, with treatment modification occurring only after LPA and DST results become available. This delay, typically lasting 4–6 weeks, hampers the timely initiation of resistance-profile directed treatment. As a result of this sub-optimal testing and treatment cascade, many patients may receive a limited number of effective drugs in the empiric regimen, increasing the risk of amplifying drug resistance and ongoing transmission of DR-TB.^3^

A further challenge hindering the diagnosis and treatment of DR-TB is the lack of susceptibility testing for INH resistance by assays available at the point where the patient enters the care cascade, including WHO-endorsed Xpert MTB/RIF, Xpert MTB/RIF Ultra or the Truenat assays. Consequently, INH mono-resistant TB patients (highly resistant TB (HR-TB) patients who display INH resistance in the absence of RR) are not appropriately diagnosed and are misdiagnosed as drug-susceptible TB (DS-TB). This subset of patients receive standard DS-TB treatment, increasing the risk of acquired resistance to other drugs in the regimen.^13^

Recommendations from the 2021 WHO DR-TB guidelines have led to a rapid transition to all-oral treatment regimens for MDR-TB patients, globally. This study attempts to address a notable research gap in implementation science by evaluating the impact of the recently endorsed Xpert XDR diagnostic to guide treatment with novel, all-oral DR-TB regimen. Implementation of shorter, efficacious and, more importantly, less toxic regimens than the conventional long regimen holds significant promise for enhancing treatment success rates. Hence, evaluation of the utility of the Xpert XDR in enhancing use of this novel regimen could help preserve the potency of new drugs by preventing rapid amplification of resistance through selection of appropriate drug components of the treating regimen.

The WHO-listed 30 high TB burden countries collectively contribute to approximately 90% of the global disease burden. South Africa and Nigeria feature on this list with significantly overlapping epidemics of TB, HIV and MDR-TB.^3^

With the emergence of newer short-course regimens, it is crucial to prioritise the early initiation of these treatment options. Evaluating tools such as the Xpert MTB/XDR for early initiation of an appropriate regimen is therefore crucial.

The GeneXpert platform is an automated cartridge-based system for TB diagnosis. Endorsed by the WHO in 2010, the Xpert MTB/RIF assay has become a cornerstone of TB programmes worldwide. Subsequently, the Xpert MTB/RIF Ultra assay replaced its predecessor, demonstrating heightened sensitivity particularly in paucibacillary disease. However, it is important to note that besides identifying RR, both the Xpert MTB/RIF and Ultra assays do not offer additional information regarding the resistance profile of the infecting strain.

The Xpert MTB/XDR assay is intended for use as a reflex test to a positive Xpert MTB/RIF or Ultra result on the 10-colour technology. The assay targets eight genes and promoter regions in *M.tb*, facilitating the detection of resistance to INH, FQs, ethionamide (ETA) and the second-line injectables. Consequently, it enables the diagnosis of pre-XDR-TB in combination with Xpert MTB/RIF or Ultra. In a preclinical study, involving 100 sputum samples and 214 clinical isolates, the Xpert MTB/XDR assay demonstrated excellent analytical performance, exhibiting a sensitivity of 94–100% and specificity of 100% for all drugs except for ETA when compared with sequencing and equivalent sensitivity and specificity when compared with phenotypic DST.^14 15^

The MTB/XDR assay incorporates the same single-step specimen processing as the Xpert MTB/RIF and Ultra and can be implemented with minimal staff training and biosafety requirements. These features are particularly significant, as they enable the potential use of the Xpert MTB/XDR assay in peripheral sectors of the global healthcare system, where rapid identification of expanded drug resistance could improve therapeutic decision-making. Notably, the WHO endorsed the use of the Xpert MTB/XDR assay for diagnosing TB drug resistance as of June 2021.^16^ However, the additional cost and technical expertise required to upgrade to the 10-colour Xpert XDR module has been prohibitive to laboratories in many disease endemic resource limited settings. Additionally, assessing appropriate placement of the Xpert XDR within the DR-TB diagnostic algorithm and adjusting laboratory workflow to accommodate this diagnostic has resulted in delays in scale-up and uptake of the Xpert XDR despite the WHO endorsement.

The focus of this study is to use the Xpert MTB/XDR assay per its intended use as a reflex test to a positive Xpert MTB/RIF or Ultra result to triage participants by promptly identify varying resistance profiles to guide the selection of appropriate treatment regimens. With optimal implementation, this new test is expected to guide the selection of appropriate regimens, thereby reducing the time to appropriate treatment for improved patient outcomes for RR-, MDR- and pre-XDR-TB.^14 17^ It is important to note that the updated pre-XDR and XDR-TB definition now warrants extended DST for BDQ, LZD and PMD to confirm extensively drug-resistant TB (XDR-TB). The Xpert MTB/XDR assay was endorsed recently by WHO, and implemented in several settings, this study will provide implementation guidance to help define best use and generate evidence to further promote uptake.^18^ In addition, the planned whole genome sequencing (WGS) and minimum inhibitory concentration (MIC) analysis in this study will assess the diagnostic and clinical performance of the Xpert MTB/XDR assay. These analyses will enable the full characterisation of drug resistance in pre-XDR-TB samples identified by the assay.

The tuberculosis molecular bacterial load assay (TB-MBLA) is a novel, quantitative reverse transcription PCR technique that uses 16S ribosomal ribonucleic acid (16S rRNA) targets to quantify the *M.tb* bacillary load in real time during treatment.^19^ A major limitation of the current TB diagnostic and management algorithm is the absence of a sensitive and rapid assay for assessing treatment efficacy and predicting treatment response. Present therapeutic monitoring in clinical samples typically infers treatment response from mycobacteria growth indicator tube (MGIT), time to a positive result (TTP) and smear microscopy. However, this approach is limited by availability of culture resources, specialised laboratory infrastructure and suitably trained personnel. Compounding these issues several factors may impact quality and consistency of TB culture results such as need for timely specimen transport, specialised culture media and need for a decontamination process to eliminate non-mycobacterial flora. Even resources, culture-based results are often not provided in time for clinical decision-making.

The TB-MBLA targets abundant 16S rRNA, enabling accurate quantification of viable *M.tb* bacillary load directly from patients’ sputum over many weeks of treatment, potentially replacing culture and offering a promising solution to improved precision in clinical care. This assay rapidly measures viable *M.tb* bacilli and their response to anti-TB therapy, enabling clinicians to make informed decisions about patient progress. Furthermore, bacillary load measured by TB-MBLA concurs with standard MGIT culture results, correlates with symptom decline^20 21^ and can accurately distinguish the bactericidal effect of different anti-TB regimens.^22^ Its high specificity, sensitivity, rapid time to results and avoidance of data loss from contamination suggest TB-MBLA could replace microscopy and culture for treatment response monitoring.^23 24^

The TB-MBLA has undergone evaluation in more than 16 high TB burden, low-resource settings countries worldwide. Data showed that TB-MBLA reduces time to detection of *M.tb* bacillary loads from weeks to hours, demonstrating reproducibility across different laboratory settings. This rapid turnaround time has the potential to inform real-time clinical decisions regarding patient treatment and expedite TB drug trials.^25^ This current study presents a unique opportunity to assess the role of TB-MBLA in monitoring treatment response in participants receiving varying DR-TB regimens.

The participation in this study offers direct benefits to patients. Participants will have access to novel drugs, diagnostics and improved treatment monitoring, which are not otherwise available through standard of care practices. We believe that the diagnostic methods and drugs used in this study could significantly improve TB treatment outcomes. Additionally, rapid diagnosis coupled with initiation of appropriate treatment has the potential to reduce the number of participants lost in the DR-TB care cascade. Generating evidence through implementation research on the feasibility, acceptability and cost-effectiveness of novel diagnostics in improving DR-TB outcomes will fast-track the translation study findings into policies and practices, maximising public health benefits. Given the development of these novel tests and the importance of a public health approach in triaging DR-TB participants to suitable all-oral regimens, a study evaluating the strategy is warranted. Finally, given the delays in DR-TB diagnosis and appropriate treatment initiation, identifying strategies to improve DR-TB outcomes is crucial for the success of TB programmes globally.

The risk benefit varies depending on the type of resistance pattern detected. Detection of mutations conferring resistance to multiple drug classes is particularly advantageous for those infected with such strains compared with those without any detectable resistance. Use of the Xpert MTB/XDR diagnostic and translation of test results for treatment modification does not preclude access to all other available diagnostic tests for DR-TB in the study settings. Until the completion of this study, it remains uncertain whether a novel diagnostic guiding the use of short-course DR-TB treatment will reduce time to appropriate treatment initiation and thereby improving treatment outcomes in DR-TB participants.

Risk of treatment-related toxicity with DR-TB medication remains consistent across all participants exposed to given DR-TB regimen. These risks include drug toxicities from DR-TB medication and drug interactions. Several new drugs in novel regimens will be made available to participants in this study from their respective DR-TB programmes. Stringent safety assessments from human trials evaluating these regimens have already been well-documented. Despite this, study participants will be closely monitored for drug toxicity. Several strategies have been implemented within this study to ensure safety of all participants. These measures include clinical observation and referral for clinical care for participants experiencing adverse events (AEs), real-time safety report to the study medical monitoring team (MMT), review of all safety reports by local ethics committees and annual review by the study’s independent Data Safety Monitoring Board (DSMB).

## Methods and analysis

### Patients and public involvement

Clinical sites have Community Advisory Boards that have reviewed the study protocol and provided input on community engagement, study planning and recruitment of participants. In addition, the study protocol has been presented at various meetings involving ministries of health from South Africa, Nigeria and Ethiopia. Patients or members of the public were not directly involved in the protocol development of the TRiAD study.

### Study setting

**Figure 1:**
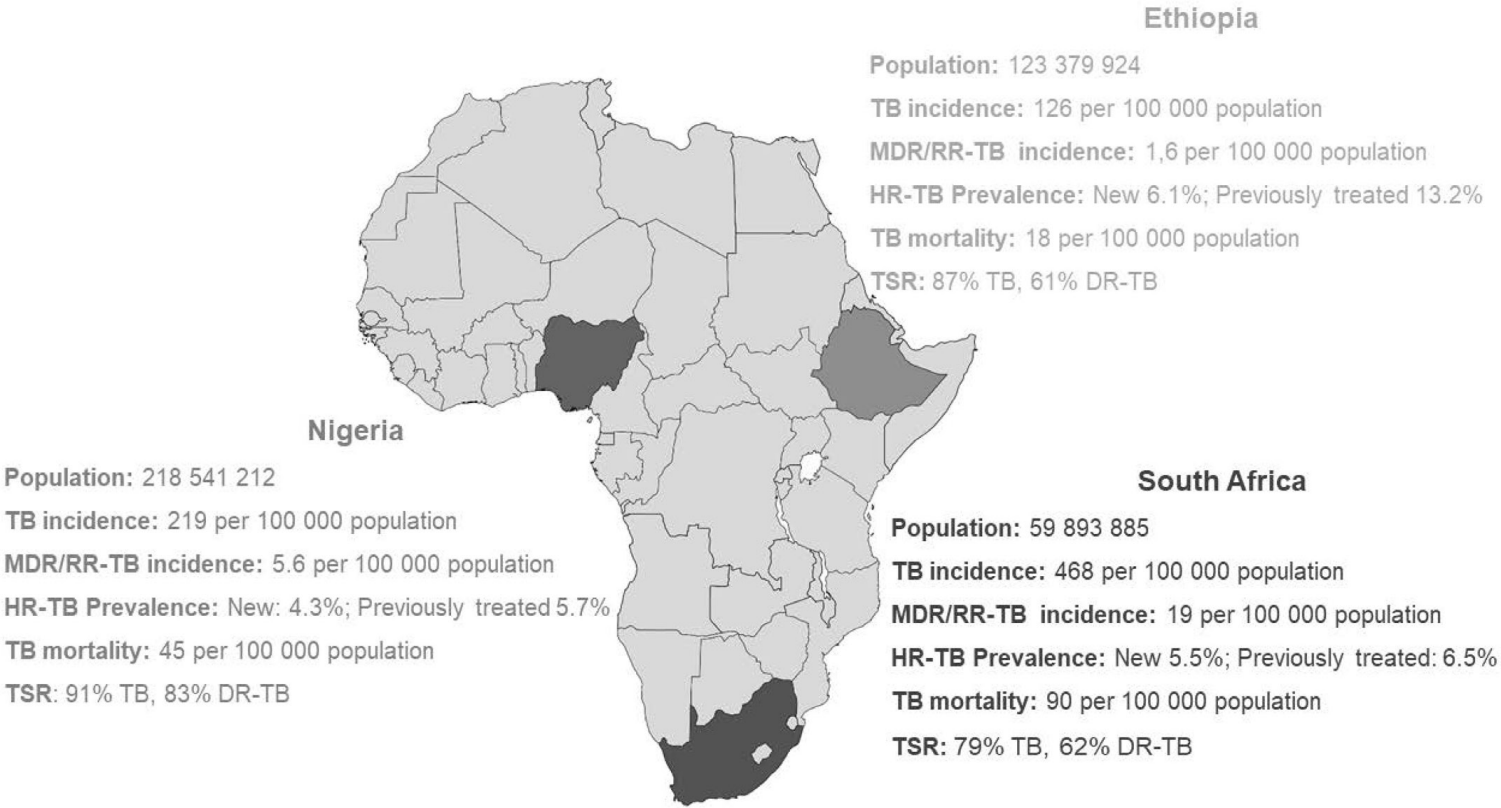
Geographical representation of three countries implementing the TRiAD study. DR-TB, drug-resistant TB; MDR/RR-TB, multi-drug-resistant/rifampicin resistance tuberculosis; HR-TB, highly resistant TB; ; TB, tuberculosis; TSR, treatment success rate.

The TRiAD study is a multinational study, conducted across multiple clinical research sites (CRS) located within South Africa, Nigeria and Ethiopia, as illustrated in figure 1. Countries were selected based on diverse representation of DR-TB prevalence, participant population, drug resistance patterns and laboratory capacities.

### Study population and selection

The TRiAD study aims to enrol 1280 adult participants: Cohort 1 will include approximately 880 RR-TB participants, and Cohort 2 will consist of approximately 400 participants who are RIF-susceptible with resistance to INH, FQ and/or SLID. Individuals who meet study eligibility criteria for each cohort and agree to participate in the TRiAD study will be enrolled (table 1).

**Table 1:** Participant inclusion and exclusion criteria for Cohorts 1 and 2.

Inclusion criteria	Exclusion criteria
Ambulant adults ≥18 years of age Newly diagnosed PTB patients receiving less than 5 days of treatment since new diagnosis: Cohort 1: < 5 days of DR-TB treatment Cohort 2: < 5 days of INH mono-resistant TB treatment preceding study entry for the current TB episode, or Sputum positive (smear and or culture) TB patients classified as failing first line treatment Any currently available nucleic acid amplification tests for drug resistance detection, Xpert MTB/RIF or Ultra changes/assay positive for *M.tb* infection with: Cohort 1: at least rifampicin resistance Cohort 2: rifampicin susceptible co-occurring with INH, FQ, ETA or aminoglycoside resistance (detected by Xpert XDR) occurring alone or in combination Capacity to provide informed consent HIV infected and uninfected participants are allowed in the study. Participants already on ART will be allowed in the study provided the ART regimen in use has no contraindications to the proposed TB drug regimen Willing to have samples collected, stored indefinitely and used for research purposes Able to provide reasonable proof of identity (to satisfaction of study team member) at or prior to enrolment	Study participants will be excluded if one or more of the following conditions apply: Has a known severe allergy to any of the BPaL component drugs Has DST showing infection with a strain resistant to any of the component drugs Has TB meningitis, other central nervous system TB or TB osteomyelitis or Is pregnant or breastfeeding Is unable to take oral medications Persons with any other medical condition, precluding study participation based on investigator judgement Any co-existing condition that in the opinion of the attending clinician renders the participant unsuitable for participation in the study Co-enrolment in other interventional research studies Late exclusion criteria Resistance to BPaL component drugs on phenotypic DST conducted post study enrolment

BPaLbedaquiline-pretomanid-linezolidDR-TBdrug-resistant TBDSTdrug susceptibility testingETAethionamide FQfluoroquinolone INHisoniazid PTBpulmonary TBTBtuberculosis

### Study design

TRiAD is a phase 4 observational prospective cohort study evaluating the effectiveness, feasibility, acceptability and cost-effectiveness of the Xpert MTB/XDR (Xpert XDR; Cepheid) assay for the rapid triage and treatment of DR-TB. The study will enrol participants and conduct active follow-up for the duration of TB treatment. Afterwards, passive follow-up will be carried out for a maximum of 18 months after study enrolment. Enrolment into the TRiAD study began in June 2022 and is projected to continue to the end of March 2024. Data collection is ongoing with a projected end date of December 2024.

### Interventions

The TRiAD study comprises three interventions: (1) the Xpert MTB/XDR assay will be used as a diagnostic adjunct to the Xpert MTB/RIF, Ultra or any currently available nucleic acid amplification tests for drug resistance detection changes to screen participants. The Xpert MTB/XDR assay will facilitate prompt identification of the following DR-TB participant categories: (a) HR-TB, (b) RIF mono-resistant TB, (c) MDR-TB and (d) additional FQ resistance, in other words, pre-XDR-TB. (2) Next is the early initiation of the most appropriate, evidence-based DR-TB regimen guided by the TB resistance pattern categorisation from the Xpert MTB/XDR diagnostic or any currently available nucleic acid amplification tests for drug-resistance detection. The choice of DR-TB regimen will be determined by: (a) the best available evidence generated from the use of novel regimens as they become available or (b) current WHO and in-country guidelines. (3) In parallel, the TB-MBLA will be used to provide real-time bacillary load monitoring throughout the treatment course to assess response to treatment. Primary and secondary objectives as well as outcomes are listed in table 2.

**Table 2:** TRiAD study objectives and endpoint

Study objectives	Study endpoints
Primary objectives	Primary endpoint
To compare the time to initiation of resistance pattern appropriate TB treatment from the date of first sputum collection with the standard of care compared with a historical cohort, stratified by geographical location To assess the proportion of DR-TB participants with cure and/or DR-TB treatment completion at month 12 compared with a historical control group	The time to appropriate treatment initiation will be calculated as median days (with IQR) between the first sputum collection date and the date of start of the regimen appropriate to the drug resistance pattern (defined as drug resistance pattern appropriate treatment). This will be compared with the same time in the historical cohort and stratified by geographical location The proportion of favourable study outcomes (treatment cure or completion, based on the WHO definitions (5)) will be calculated in the overall study population of Cohort 1, and compared with the same proportion in the historical cohort, and stratified by geographical location
Clinical	
To assess the safety and effectiveness of all-oral regimens under operational conditions. To assess all-cause mortality at months 12 and 18, rates of drug/regimen discontinuation for safety concerns and rates of drug-related AEs	The incidence of adverse events (AEs) will be divided into AEs and serious AEs (SAEs) and calculated in proportions using both Cohorts 1 and 2. These will also be stratified by type of DR-TB, treatment regimen and HIV status
Microbiological	
To assess time to culture conversion, cure and rates of clinical TB relapse To determine the prevalence of HR-TB stratified by geographic location To determine the prevalence of pre-XDR and XDR-TB using phenotypic DST to BDQ, PMD and LZD To determine the proportion of participants with BDQ and LZD resistance warranting a switch from short-course treatment To assess the clinical utility of the TB molecular bacterial load assay (TB-MBLA) as a predictor of treatment outcome To assess the diagnostic and clinical performance of the test and triage approach by geographic location using WGS and MIC analysis To determine the phylogenetic structure and full drug resistance patterns of study samples, including BDQ, LZD, levofloxacin (LFX) and PMD	Accuracy of Xpert XDR testing (in comparison with culture+DST/LPA/next-generation sequencing (NGS)), by type of DR-TB (Cohorts 1 and 2) Clinical utility and feasibility of TB-MBLA in bacteriological follow-up of DR-TB (in comparison with routine culture) by type of DR-TB and regimen (Cohort 1) Quality of Xpert XDR tests (indeterminate rate, DNA contamination rate and performance variation (in time and between sites)) (Cohorts 1 and 2) Mathematical modelling of TB-MBLA trajectories in response to treatment Prevalence of HR-TB stratified by geographical location. This will be calculated in Cohort 2 and stratified by HIV status Additional resistance to new and repurposed drugs, by type of DR-TB and regimen (Cohorts 1 and 2)
Cost-effectiveness, feasibility and acceptability compared with the standard of care	
To determine cost per person tested and incremental cost per DR-TB participant timely initiated on the resistance- pattern appropriate regimen To determine the incremental cost per disability-adjusted life year averted and per day delay averted in treatment with the resistance pattern appropriate regimen	Cost-effectiveness (cost per person tested and incremental cost per DR-TB participant timely initiated on the resistance pattern-appropriate regimen) and, following from that, optimal placement of Xpert XDR in diagnostic and treatment algorithm (Cohorts 1 and 2)
To assess programmatic cost-effectiveness, feasibility and acceptability of implementing Xpert MTB/RIF Ultra with Xpert MTB/XDR testing for HR and second-line DR-TB drugs To investigate participant’s, healthcare provider’s and policy-maker’s acceptability of the triage and treat approach	Feasibility and acceptability (participant and healthcare provider perspective), stratified by type of DR-TB regimen (Cohorts 1 and 2)
To assess the feasibility of the implementation of the triage and treat approach in routine care	Operational feasibility of Xpert XDR testing (infrastructure requirements and HR needs) (Cohorts 1 and 2)

BDQbedaquiline DR-TBdrug-resistant TBDSTdrug susceptibility testingHR-TBhighly-resistant TBLPAHain Line Probe AssayLZDlinezolid MICminimum inhibitory concentrationPMDpretomanid Pre-XDRpre-extensively drug-resistantTBtuberculosisWGSwhole genome sequencingXDR-TBExtensively drug-resistant TB

### Informed consent

Written informed consent will be obtained from each enrolled study participant. Additionally, written informed consent will be obtained for long-term specimen storage and potential future analyses. The informed consent procedure will be conducted in either English or the respective local languages of each country based on participant preference. In cases where a participant is illiterate, an impartial witness will be present throughout the informed consent procedure to ensure that all questions are answered to the satisfaction of the potential participant.

### Screening and enrolment

In the TRiAD study, two screening strategies will be adopted: (a) patients with suspected pulmonary TB (PTB) or confirmed *M.tb* positivity less than 5 days since treatment initiation will be screened and consented to provide an additional sputum sample for Xpert MTB/XDR testing, if required where testing cannot be done on initial sample. This will be based on the routine sample collected for any currently available nucleic acid amplification tests for drug resistance detection such as Xpert MTB/RIF or Ultra testing. Alternatively, (b) all patients with newly identified RR-TB will be concurrently contacted through laboratory records during the study enrolment period for direct participation in the study. Eligibility of potential participants will be assessed to ensure they meet the inclusion criteria stipulated by the study’s requirements. The Xpert MTB/XDR assay will be implemented as an additional DR-TB screening test.

All participants must test positive for *M.tb*. Those diagnosed with RR-TB will undergo further screening for additional resistance and thereafter enrolled in Cohort 1. Patients identified to be without RR-TB will be screened for INH resistance and subsequently enrolled into Cohort 2. Participants with HR-TB will be enrolled in Cohort 2. As TriAD is an operational study, accrual of participants into the study will be within a specified timeframe.

Participant accrual at all sites will occur over 12 to 18 months, starting from the date of enrolment of the first study participant. Participants deemed ineligible according to the exclusion criteria of the study will be referred to the National Tuberculosis Programme for further management. Furthermore, participants will be interviewed to collect demographic details and medical history (including prior TB diagnoses and history of previous TB drug intake) and undergo a targeted clinical examination conducted by a designated medical professional.

**Figure 2:**
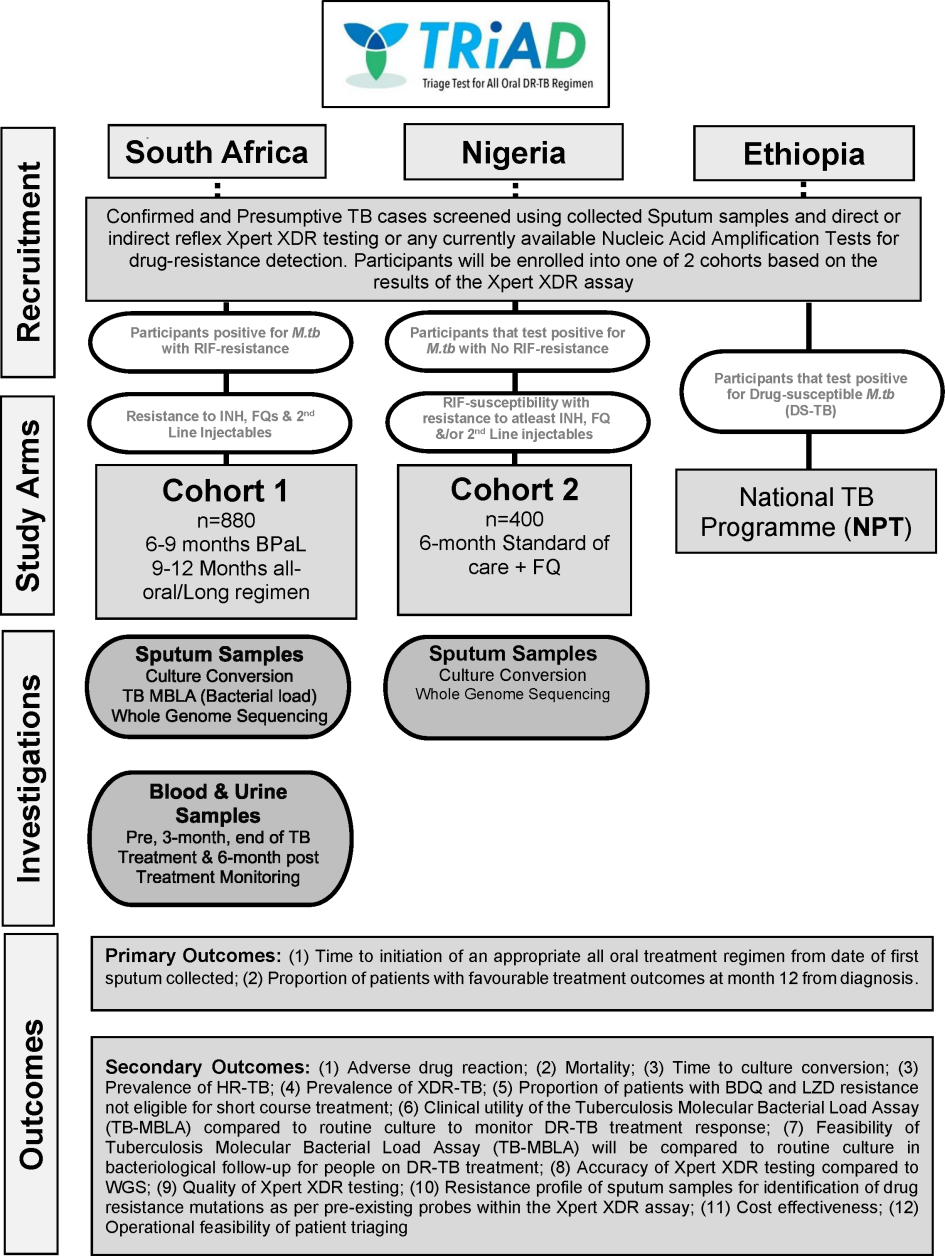
Schematic overview of the trial organisation implemented in the TRiAD study. BDQ, bedaquiline; BPaL, bedaquiline-pretomanid-linezolid; DR-TB, drug-resistant TB; FQ, fluoroquinolone; HR-TB, highly resistant TB; INH, isoniazid; LZD, linezolid; *M.tb*, *Mycobacterium tuberculosis*; RIF, rifampicin; TB, tuberculosis; XDR-TB, extensively drug-resistant TB.

The overview of TRiAD trial organisation, as adopted by the study, is outlined in figure 2.

### Follow-up

#### Safety monitoring: adverse events and serious adverse events assessment

At every follow-up visit, all study participants will be assessed for any AEs and serious AEs (SAEs). The guidelines used to grade the severity of adverse drug reactions (ADRs) are provided (online supplemental material 1, table 1). The principal investigator (PI) or designee appointed by the PI must determine the severity of the AE and be responsible for documenting the incidence on the appropriate case report form. Ongoing AEs at the time of study exit will be followed up for a period of up to 30 days. If not resolved, the patient will be referred to a healthcare provider for further intervention.

In cases of toxicity with any of the drugs used in the study, the decision on whether to continue or stop the drug depends on factors such as severity of toxicity exhibited by the participant, duration of treatment received, culture conversion status, number of other active drugs included in the regimen, the essential nature of the offending drug to the regimen and the possibility of replacing it with another active drug. Local DR-TB programme management guidelines will be followed for all adverse events detected.

The TRiAD study has a designated international MMT available to directly discuss AEs with sites, offer advice to investigators on patient safety and provide oversight on any general safety concerns that arise during the trial, ensuring they are addressed in real time.

### Statistical analysis

#### Power and sample size calculations

The sample size and power calculation were derived from the two primary endpoints, exclusively focusing on Cohort 1: (i) identifying the requisite numbers to discern a reduction in the time taken to initiate treatment appropriate to the resistance pattern for all cases of DR-TB compared with the historical cohort and (ii) assessing the disparity in the proportion of favourable treatment outcomes compared with the historical cohort, with stratification by study site, particularly geographical location. The primary outcome will be computed solely in Cohort 1, as reliable historical data are unavailable for either endpoint for HR-TB (Cohort 2). Data observed mainly from South Africa exhibited median time to resistance pattern appropriate treatment initiation for drug-resistant participants between 15 and 46 days.^1 3 26^ Assuming an anticipated time-to-treatment initiation of 1 week (additional sputum samples and delays in Xpert testing), 220 RR-TB participants must be enrolled in Cohort 1 in each country to achieve 90% power to show a shortening of time to treatment initiation. This sample size includes an allowance for an overall 15% loss to follow-up and analysis adjusted for clustering by study site. The time taken for appropriate treatment initiation in Cohort 1 will be determined as the interval between the first sputum collection date and the commencement of the regimen deemed suitable for the drug resistance pattern. Median and IQR summaries will be juxtaposed with those of the historical cohort (Cohort 3), stratified by geographical location. Cox regression will be employed for time-to-event analysis, with adjustment for confounding factors through multivariable analysis and clustering by study site. The proportion of favourable outcomes in Cohort 1 will be compared with the corresponding proportion in Cohort 3, stratified by geographical location. Secondary analysis of these proportions will include calculations per type of DR-TB and by study site. Multivariable logistic regression analysis will be used for proportion comparison, incorporating any potential confounding variables. It is imperative to acknowledge that random effects analysis will be employed to accommodate clustering by study site, and calendar time will be incorporated to adjust for any period effects between Cohorts 1 and 3.

#### Data safety monitoring and participant review

The DSMB acts independently to monitor participant safety, study progress and outcomes. The responsibilities of the DSMB include evaluating study progress and conducting periodic participant safety assessments. The DSMB members are independent of the study team, have no direct interest in the study outcome and all possess significant experience in DR-TB research. These members were selected based on their willingness to disclose financial interests and declare conflicts of interest at each meeting.

Day-to-day primary safety oversight of trial participants is provided by the MMT. The MMT responds to queries in real time and reviews safety reports at regular MMT meetings. Meeting minutes from the MMT are shared with the study statistician and DSMB. Additionally, all safety concerns raised by implementing staff with the MMT are summarised for the DSMB.

#### Data management

All data management activities will be conducted in compliance with applicable regulatory frameworks and managed by CAPRISA for all sites. This includes the European Medicines Agency regulations concerning data integrity, data protection and privacy, as well as compliance with the relevant health products regulatory authorities of South Africa, Nigeria and Ethiopia. The TRiAD study will adhere to all pertinent institutional review board regulations. CAPRISA’s data management systems meet the Food and Drug Administration (FDA) requirements, as they are compliant with Code of Federal Regulations (CFR) Part 11 (the segment of Title 21 of the CFR that outlines the US FDA regulations on electronic records and electronic signatures), and the data management standard processes are aligned with the Good Clinical Data Management Processes.

#### Cost-effectiveness

Through cost-effectiveness analyses, the TRiAD study will determine the incremental cost per disability-adjusted life year averted through the implementation of combined Xpert MTB/RIF or Ultra and Xpert/XDR, followed by an all-oral, STR compared with the conventional standard of care for DR-TB patients. The cost-effectiveness comparison between the intervention and SOC will be modelled using previously published models of cost-effectiveness on the GeneXpert framework.^27^,^29^ The timeframe of the model-based analysis will span from initial diagnosis to the end of life. Deterministic and probabilistic sensitivity analyses will be performed on model parameters and methodological assumptions.

### Feasibility and acceptability

To assess the acceptability and feasibility of the new triaging and treatment algorithms, a mixed methods study design will be used. Quantitative data will be gathered through prestructured questionnaires, while in-depth interviews will complement these results. Participants and healthcare workers (HCWs) from each CRS and one policy-maker from each country will be recruited with a focus on variability in gender, regimens, comorbidities, adherence status, and HCWs and stakeholders with diverse job descriptions and experience levels. Qualitative data collection will continue until saturation, though only one policy-maker will be interviewed per level. Quantitative data analysis will use statistical software such as Stata or SPSS, and qualitative data will be analysed using QSR Nvivo10 software with an applied thematic approach. A coding scheme will be developed and refined through regular discussions among researchers. Study results will be stratified by country, participant/HCW perspective, gender and regimen type and presented by themes with relevant interviewee quotes. It is important to note that in this study, acceptability is defined by cognitive and emotional responses to the intervention, while feasibility concerns its practical implementation. Both will influence the intervention’s uptake and effectiveness.

## Ethics and dissemination

The University of KwaZulu-Natal Biomedical Research Ethics Committee (BREC) and local research committees have provided ethical review and approval (BREC/00002654/2021, Wits HREC 210805, NHREC/01/01/2007 and EPHI-IRB-459–2022). In addition, the South African Health Products Regulatory Authority (SAHPRA) have granted regulatory approval for use of Xpert XDR in South Africa (SAHPRA MD20211001). The study has been registered with Clinicaltrials.gov (registration number; trial registration number: NCT05175794). Trial results will be disseminated through conference presentations, peer-reviewed publications and the clinical trial registry.

## Conclusion

The TRiAD study will provide real-world operational insights on the utility of the Xpert XDR in guiding novel all-oral DR-TB regimen use in patients with DR-TB. This implementation trial provides opportunities to assess the effectiveness, feasibility, acceptability and cost-effectiveness of the Xpert XDR to efficiently triage patients with DR-TB to receive appropriate drugs and to guide appropriate placement of the XPERT XDR assay in the TB testing algorithm, that is, to be used in parallel to or as a reflex test to test for *M.tb* detection; the sensitivity and specificity of Xpert XDR in detection of IND, FQ, ETA and SLID resistance in various geographic settings; the use of the mycobacterial load assay as a treatment response marker in DR TB and the impact of the novel diagnostics in improving patient outcomes. This study will provide valuable operational insights that will shape scalability and sustainability of these novel diagnostics and will influence DR-TB diagnostic and therapeutic guidelines including providing direction on future research endeavours.

## supplementary material

10.1136/bmjopen-2024-084722 online supplemental file 1
