# Supplementary material for: Triage test for all-oral drug-resistant tuberculosis (DR-TB) regimen: a phase IV study to assess effectiveness, feasibility, acceptability and cost-effectiveness of the Xpert MTB/XDR assay for rapid triage and treatment of DR-TB
Source: BMJ Open. 2024 Nov 27;14(11):e084722. doi: 10.1136/bmjopen-2024-084722 (PMC11603726; doi:10.1136/bmjopen-2024-084722)
Supplement: online supplemental file 1 [file bmjopen-14-11-s001.pdf]

Table 1: Grading the severity of adverse drug reactions; following common guidelines and management for the treatment of TB used in the TRiAD study.

Grade	Severity Rating	Definition
**Grade 1**	Mild	Transient or mild discomfort (< 48 hours); no medical intervention/therapy required. The participant will be followed carefully, and the study drugs will be continued
**Grade 2**	Moderate	Mild to moderate limitation in activity - some assistance may be needed; no or minimal medical intervention/therapy required. The participant will be followed more closely, with additional laboratory and/or clinic visits as necessary; treatment should be continued unless in the view of the clinician/investigator this would be unsafe.
**Grade 3**	Severe	Marked limitation in activity, some assistance usually required; medical intervention/therapy required, hospitalizations possible.
**Grade 4**	Potentially life threatening	Extreme limitation in activity, significant assistance required; significant medical intervention/therapy required, hospitalization or hospice care probable.
**Grade 5**	Fatal	Death
