# Supplementary material for: Triage test for all-oral drug-resistant tuberculosis (DR-TB) regimen: a phase IV study to assess effectiveness, feasibility, acceptability and cost-effectiveness of the Xpert MTB/XDR assay for rapid triage and treatment of DR-TB
Source: BMJ Open. 2024 Nov 27;14(11):e084722. doi: 10.1136/bmjopen-2024-084722 (PMC11603726; doi:10.1136/bmjopen-2024-084722)
Supplement: Supplementary file 2 [file bmjopen-2024-084722.reviewer_comments.pdf]

PEER REVIEW HISTORY

BMJ Open publishes all reviews undertaken for accepted manuscripts. Reviewers are asked to complete a checklist review form and are provided with free text boxes to elaborate on their assessment. These free text comments are reproduced below.

ARTICLE DETAILS

Title (Provisional)

The changing nature of worry about COVID-19 among the English public: A secondary analysis of 73 national, cross-sectional surveys, January 2020 to April 2022.

Authors

Rubin, G James; Smith, Louise E.; Amlôt, Richard; Fear, Nicola T; Potts, Henry; Michie, Susan

---

VERSION 1 - REVIEW

**Reviewer**	**1**
**Name**	**Wu, Jian**
**Affiliation**	**Zhengzhou University**
**Date**	**02-Jul-2024**
**COI**	**No**

The manuscript explored how worry about COVID-19 evolved during the pandemic and attempted to correlate public worry with multiple factors. Several significant issues need to be addressed.

1. The manuscript lacks a clear explanation of the survey methodology, including sampling strategies, survey schemes, and response rates. Without this information, it is difficult to assess the reliability and validity of the findings.

2. The questionnaire items are simple and lack rigor. In addition, participants' self-answers are easily influenced by subjective cognition. For example, if the participants themselves suffer from anxiety and depression, they will exaggerate their concerns about the novel coronavirus epidemic.

3. There is no discussion of potential biases, particularly selection bias, given that participants were drawn from an online panel. The representativeness of this sample concerning the broader English population is questionable.

4. The author has an insufficient understanding of the source of the COVID outbreak, and the naming of the "Wuhan coronavirus" lacks preciseness.

5. The analysis appears to be overly descriptive, lacking rigorous statistical testing to substantiate the claims. The manuscript would benefit from more sophisticated statistical analyses.

6. The clarity of the drawings in the manuscript is not high, and it is suggested that the author revise them.

**Reviewer**	**2**
**Name**	**Raude, Jocelyn**
**Affiliation**	**EHESP Rennes-Sorbonne Paris Cité**
**Date**	**17-Jul-2024**
**COI**	**None**

This is a well-written article investigating the dynamic of worry about an infectious disease, which addresses an important and somewhat neglected topic in the current literature on health behaviors. The authors used sound data collected among large and representative samples of the UK population in a repeated measures research design to examine the association between certain affective-cognitive variables and social-epidemiological variables over time. However, there are in my view a number of conceptual, methodological and theoretical weaknesses that should be addressed before considering the paper for publication in BMJ open.

1. Conceptual issues:

In my understanding, worry is not exactly the same as anxiety, but these terms are used interchangeably in the article. According to the APA dictionary, anxiety refers to “an emotion characterized by apprehension and somatic symptoms of tension in which an individual anticipates impending danger, catastrophe, or misfortune”, while worry refers to “a state of mental distress or agitation due to concern about an impending or anticipated event, threat, or danger”. Based on the APA definitions, only a persistent and excessive worry should be considered a main symptom of anxiety disorder. In general, clinical psychologists make a clear distinction between anxiety and fear as the former is considered “a future-oriented, long-acting response broadly focused on a diffuse threat”, whereas the latter is “an appropriate, present-oriented, and short-lived response to a clearly identifiable and specific threat”. Even though the authors asked the participants about their worry about coronavirus, I wonder if the term "fear" might be more appropriate than "worry" or "anxiety" in this article.

2. Methodological issues:

The association between concern and socio-epidemiological characteristics (COVID mortality rate, government intervention) is analyzed only graphically. This is an important first step, but in my opinion the demonstration deserves a more detailed statistical analysis of the relationships between these variables. Given the nested structure of data collected through repeated cross-sectional surveys, the authors should use multilevel models to estimate survey effects simultaneously with the effects of the survey-level predictors, such as the COVID-19 mortality rate. A minor comment: the incidence rates reported by the authorities does not represent a reliable measure of the COVID-19 dynamic as there was only a very limited testing capacity during the early phase of the pandemic.

3. Theoretical issues:

The authors should pay be more attention to issues related to knowledge cumulativity. Unexpectedly, no theoretical element was presented to account for the temporal pattern of worry identified in the study. The authors should discuss their main results in the light of a range of possible psychological effects/mechanisms highlighted in previous research in the field of infectious diseases, such as risk adaptation (Loewenstein & Mather, 1990), risk reappraisal (Brewer et al, 2004) or risk habituation (Raude et al, 2019). For a review, see Martin-Lapoirie et al (2024). Moreover, the results obtained in this research should be further discussed in the light of other studies carried out in comparable countries on the same topic, especially as these results are relatively convergent with those of the authors (for instance, Lee et al in South Korea in PLoS One).

**Reviewer**	**3**
**Name**	**Eraso, Yolanda**
**Affiliation and Professions**	**London Metropolitan University, School of Social Sciences**
**Date**	**23-Jul-2024**
**COI**	**No competing interests**

Thank you for the opportunity to review this manuscript. The research appears comprehensive and well-structured, drawing on a unique dataset of 73 surveys to explore levels of worry in the English population and its potential associations during the COVID-19 pandemic (Jan 2020-April 2022). The figures to represent key findings or trends are very well developed, given the amount of information to synthesise. There are, however, some suggestions for improvement:

Keywords: it contains the term ‘Health communication’ and I wonder if this is a relevant term given that the article does not specifically discuss this factor.

Introduction: It would be meaningful to provide an overview of why worry at population level is important in a pandemic, given that is the focus of this paper although it has not been explicitly addressed. There is a positive/useful worry that can result in preventative behaviour, i.e. when individuals can think constructively about the risks and dangers and how to anticipate and counteract them. And there is another worry that can manifest an anxiety disorder – this is concerning as a consequence of a pandemic, but also as a concern in terms of the behavioural responses it may induce (health risks and survival) if help-seeking is affected. There is a spectrum of worry that seems collapsed here into one emotional response.

Discussion: This section needs to be strengthened to support the findings from this study. The discussion should include a thorough analysis and comparison of the results with other studies. Below are two examples:

Ethnic minority groups were more likely to be worried compared to white groups throughout the pandemic, which seems a significant finding. If worry about Covid-19 is higher and constant for this group, I wonder what the explanation might be. For example, the authors explain that for 60+ groups, the level of worry declined after vaccination was introduced in December 2020. We know from the literature that this is not the case for Black and Asian groups where vaccine hesitancy was more prevalent. What factors, then, might explain this finding?

You have measured different SEC variables (Economic hardship, deprivation, employment status and socio-economic grade) that appear to have no association with worry during the pandemic. This is different from findings from a systematic review where financial strain predicts anxiety symptoms during Covid-19. See BMC Psychol. 2024 Apr 26;12(1):237. doi: 10.1186/s40359-024-01715-8. Also observed in a UK study: BJPsych Open. 2020;6(6):e125. doi:10.1192/bjo.2020.109

The discussion could also be expanded to include potential policy implications (pandemic preparedness), and recommendations for further research.

VERSION 1 - AUTHOR RESPONSE

Dear Dr Martin

We are grateful to the reviewers for having taken the time to look at our manuscript. We have now revised it according to their suggestions and have responded to these below.

Reviewer: 1

Prof. Jian Wu, Zhengzhou University

Comments to the Author:

The manuscript explored how worry about COVID-19 evolved during the pandemic and attempted to correlate public worry with multiple factors. Several significant issues need to be addressed.

1. The manuscript lacks a clear explanation of the survey methodology, including sampling strategies, survey schemes, and response rates. Without this information, it is difficult to assess the reliability and validity of the findings.

A full account of the survey methods is available in Smith LE, Potts HWW, Amlôt R, Fear NT, Michie S, Rubin GJ. Adherence to the test, trace, and isolate system in the UK: Results from 37 nationally representative surveys. BMJ. 2021;372. We have added an explicit link to this paper to the design section of methods section for readers interested in the detailed account.

2. The questionnaire items are simple and lack rigor. In addition, participants' self-answers are easily influenced by subjective cognition. For example, if the participants themselves suffer from anxiety and depression, they will exaggerate their concerns about the novel coronavirus epidemic.

Our approach to measuring worry does inevitably rely on self-report. To our knowledge, there is no objective measure of worry that can be applied rapidly to a large sample of the population and as such self-report is as good as it gets for this purpose. However, we have included a statement in the limitations section that "The outcome measure was a single item, the reliability and validity of which are unclear. In terms of reliability, it may be that a scale would have provided a measure with greater reliability than a single item."

3. There is no discussion of potential biases, particularly selection bias, given that participants were drawn from an online panel. The representativeness of this sample concerning the broader English population is questionable.

This issue is addressed in our discussion section, where we note that "The data were generated from a series of cross-sectional online surveys with participants drawn from an existing panel of people interested in responding to surveys on a wide range of topics in return for compensation. The representativeness of such samples is not clear, although the fact that participants did not specifically volunteer because the survey related to the pandemic reduces the risk of bias related to interest in the topic (26)."

4. The author has an insufficient understanding of the source of the COVID outbreak, and the naming of the “Wuhan coronavirus” lacks preciseness.

The use, by the English Department of Health and Social Care, of the term “Wuhan coronavirus” in their early surveys reflected a desire to ensure that the lay respondents to the survey understood what the survey related to. This was the term that had been used by some sections of the English media. We agree entirely that the phrase is unfortunate, but it is what DHSC used and therefore we must report it as such. We have clarified this in the Study measures section of the methods (“Up until wave 5, the surveys referred to ‘Wuhan coronavirus’ as this term had been used by some section of the media in England.”)

5. The analysis appears to be overly descriptive, lacking rigorous statistical testing to substantiate the claims. The manuscript would benefit from more sophisticated statistical analyses.

Please see our response to Reviewer 2, point 2a.

6. The clarity of the drawings in the manuscript is not high, and it is suggested that the author revise them.

We apologise for this – we uploaded clear figures to the system, but these may have become altered somehow as the system turned them into PDFs. We will work with the journal’s editorial team to ensure the final figures are of higher quality.

Reviewer: 2

Dr. Jocelyn Raude, EHESP Rennes-Sorbonne Paris Cité

Comments to the Author:

This is a well-written article investigating the dynamic of worry about an infectious disease, which addresses an important and somewhat neglected topic in the current literature on health behaviors. The authors used sound data collected among large and representative samples of the UK population in a repeated measures research design to examine the association between certain affective-cognitive variables and social-epidemiological variables over time. However, there are in my view a number of conceptual, methodological

and theoretical weaknesses that should be addressed before considering the paper for publication in BMJ open.

1. Conceptual issues:

In my understanding, worry is not exactly the same as anxiety, but these terms are used interchangeably in the article. According to the APA dictionary, anxiety refers to “an emotion characterized by apprehension and somatic symptoms of tension in which an individual anticipates impending danger, catastrophe, or misfortune”, while worry refers to “a state of mental distress or agitation due to concern about an impending or anticipated event, threat, or danger”. Based on the APA definitions, only a persistent and excessive worry should be considered a main symptom of anxiety disorder. In general, clinical psychologists make a clear distinction between anxiety and fear as the former is considered “a future-oriented, long-acting response broadly focused on a diffuse threat”, whereas the latter is “an appropriate, present-oriented, and short-lived response to a clearly identifiable and specific threat”. Even though the authors asked the participants about their worry about coronavirus, I wonder if the term "fear" might be more appropriate than "worry" or "anxiety" in this article.

This is a tricky issue. On balance, because the item is worded as “how worried are you” we think we should stick to referring to it as a measure of worry in the main text. However, we think Dr Raude is right that it is not clear-cut and have therefore added this statement to the limitations section of the discussion: “In terms of validity, although the item specified “how worried are you,” we do not know whether responses were more affected by worry, or the related but separate concepts of fear or anxiety.”

2a. Methodological issues:

The association between concern and socio-epidemiological characteristics (COVID mortality rate, government intervention) is analyzed only graphically. This is an important first step, but in my opinion the demonstration deserves a more detailed statistical analysis of the relationships between these variables. Given the nested structure of data collected through repeated cross-sectional surveys, the authors should use multilevel models to estimate survey effects simultaneously with the effects of the survey-level predictors, such as the COVID-19 mortality rate.

Generalised estimating equations (GEEs) are similar to multilevel models, but can be more robust to misspecification of the model (Vagenas & Totsika, 2018, https://doi.org/10.1016/j.ridd.2018.04.010). Multilevel modelling is good when fitting a

random effects model. While we do expect a wave effect (as a proxy of a time effect), this should be modelled with a fixed effects approach here (ibid.), which we can effectively do within the GEE. The GEE also allows for the more complicated expected effect on covariance of the sampling framework. Namely, some individuals responded in more than one wave and we expect an individual's responses at different times to be more alike. The GEE models this in a manner not entirely dissimilar to multilevel modelling by empirically fitting a covariance matrix.

We have focused our statistical testing on wave as we can be sure of the wave dates. We graphically explored the possible relationship with other factors, like COVID-19 mortality rate, but it is not clear to us how that should be best included in a model. Some sort of weighted model of daily figures would be needed, possibly with a lagged term, but the exact details would require lengthy exploration. We have opted not to get into this further in this paper. We are working on another paper that compares survey results to a number of other longitudinally-collected data sets

2b. A minor comment: the incidence rates reported by the authorities does not represent a reliable measure of the COVID-19 dynamic as there was only a very limited testing capacity during the early phase of the pandemic.

We agree and have noted in the figure captions that “Case numbers before June 2020 and in April 2022 are an underestimate as widespread testing was not implemented at this time.”

3. Theoretical issues:

The authors should pay be more attention to issues related to knowledge cumulativity. Unexpectedly, no theoretical element was presented to account for the temporal pattern of worry identified in the study. The authors should discuss their main results in the light of a range of possible psychological effects/mechanisms highlighted in previous research in the field of infectious diseases, such as risk adaptation (Loewenstein & Mather, 1990), risk reappraisal (Brewer et al, 2004) or risk habituation (Raude et al, 2019). For a review, see Martin-Lapoirie et al (2024). Moreover, the results obtained in this research should be further discussed in the light of other studies carried out in comparable countries on the same topic, especially as these results are relatively convergent with those of the authors (for instance, Lee et al in South Korea in PLoS One).

We have now drawn on (in the introduction) the Lee et al and Martin-Lapoirie et al studies, and raise the issues of risk adaptation and habituation. We note that:

• “Evidence that fluctuations in the number of cases within a pandemic, and hence the level of risk, are associated with changing levels of concern suggest that people ‘adapt’ their risk perceptions to fit the changing context (6, 8).”

• “The degree to which people show progressively smaller emotional responses to similar levels of infection risk over time (‘habituation’) is still uncertain (8).”

We have also amended the discussion to highlight more explicitly the role of adaptation and habituation and how our evidence aligns with these theories. We now say:

• “While levels of worry appeared to climb as national case numbers increased throughout the pandemic, the strength of the association decreased over time. The strong associations we found between worry and perceived risk to self or others suggests that it may have been reductions in perceived risk that drove reductions in worry Changes in the level of risk to members of the public occurred throughout the period, and the reduced emotional response probably reflected a rational adaptation to the changing context (8), at least in part.”

• “The reduced impact on worry of successive waves of infection might also have been caused by habituation among the public to the risk associated with COVID-19, something that has been observed before in relation to infectious and terrorism-related threats (6, 25, 26), and has been reported elsewhere in relation to the COVID-19 pandemic (8, 27) However, if any such habituation did occur, it was not apparent for members of the public who were in clinical ‘at risk’ groups during the winter 2020/21 spike in infections. Worry within that group remained high throughout the first period of the pandemic, while during the December 2020 spike in infections worry in those who were clinically at risk returned to levels close to those seen in March 2020. If habituation to risk is a valid phenomenon, there appear to be important individual differences at play that determine who is affected by it.”

Reviewer: 3

Dr. Yolanda Eraso, London Metropolitan University

Comments to the Author:

Thank you for the opportunity to review this manuscript. The research appears comprehensive and well-structured, drawing on a unique dataset of 73 surveys to explore levels of worry in the English population and its potential associations during the COVID-19 pandemic (Jan 2020-April 2022). The figures to represent key findings or trends are very well developed, given the amount of information to synthesise. There are, however, some suggestions for improvement:

Keywords: it contains the term ‘Health communication’ and I wonder if this is a relevant term given that the article does not specifically discuss this factor.

We have removed this keyword.

Introduction: It would be meaningful to provide an overview of why worry at population level is important in a pandemic, given that is the focus of this paper although it has not been explicitly addressed. There is a positive/useful worry that can result in preventative behaviour, i.e. when individuals can think constructively about the risks and dangers and how to anticipate and counteract them. And there is another worry that can manifest an anxiety disorder – this is concerning as a consequence of a pandemic, but also as a concern in terms of the behavioural responses it may induce (health risks and survival) if help-seeking is affected. There is a spectrum of worry that seems collapsed here into one emotional response.

We have clarified in the introduction that “Outbreaks of emerging infectious diseases can cause high levels of anxiety and worry among the public. This can motivate people to take protective action (1), and influence economically relevant behaviours (2) and mental health (3). High levels of worry and anxiety are not inevitable, and several factors have been proposed as influencing a population’s emotional response to an infectious disease outbreak. Understanding these factors can help those tasked with communicating with the public to understand how best to develop their messages, providing reassurance or motivation if required.”

Discussion: This section needs to be strengthened to support the findings from this study. The discussion should include a thorough analysis and comparison of the results with other studies. Below are two examples:

Ethnic minority groups were more likely to be worried compared to white groups throughout the pandemic, which seems a significant finding. If worry about Covid-19 is higher and constant for this group, I wonder what the explanation might be. For example, the authors explain that for 60+ groups, the level of worry declined after vaccination was introduced in December 2020. We know from the literature that this is not the case for Black and Asian groups where vaccine hesitancy was more prevalent. What factors, then, might explain this finding?

We have clarified in the discussion that we believe that “The higher rates of worry among people from non-white minoritised ethnic communities that we observed, particularly

among Asian and British Asian respondents, was apparent even in January 2020, suggesting that it reflected more generalised anticipation or concern about the likely impact of the pandemic for those communities. It is possible that this links to preexisting and continuing low levels of trust in the Government among these communities, that affects the perception that the Government will take adequate steps to protect these communities during a crisis (28)."

You have measured different SEC variables (Economic hardship, deprivation, employment status and socio-economic grade) that appear to have no association with worry during the pandemic. This is different from findings from a systematic review where financial strain predicts anxiety symptoms during Covid-19. See BMC Psychol. 2024 Apr 26;12(1):237. doi: 10.1186/s40359-024-01715-8. Also observed in a UK study: BJPsych Open. 2020;6(6):e125. doi:10.1192/bjo.2020.109

Although the survey dataset does contain many sociodemographic variables, including socioeconomic grade, we did not set an a priori hypothesis relating to the link between this variable and worry, and did not present any results relating to it. We are not sure that the comparison with analyses in the cited studies that explore symptoms of anxiety is entirely valid, given that our outcome was a measure of worry, rather than symptoms of anxiety.

The discussion could also be expanded to include potential policy implications (pandemic preparedness), and recommendations for further research.

We have added an additional paragraph to the discussion to address this:

"In terms of practical implications, our data suggest that in any future pandemic it is likely that the initial spikes in population worry that will accompany the first infections or deaths within a country will wane over time. Given the importance of risk perception in driving behaviour change, this decline in worry may have implications for the maintenance of various behaviours that have health, social or economic significance. If it is correct that official advertising based on fear-appeals does little to affect this, then this suggests that public health officials who wish to encourage behaviour change should seek out other ways of doing so. With respect to research implications, we suggest that closer examination of the role of individual differences in determining habituation or adaptation to risk may be useful, given our finding those most at risk from COVID-19 appeared to maintain high levels of worry throughout the initial months of the pandemic."

VERSION 2 - REVIEW

**Reviewer**	**2**
**Name**	**Raude, Jocelyn**
**Affiliation**	**EHESP Rennes-Sorbonne Paris Cité**
**Date**	**24-Sep-2024**
**COI**	**None**

The authors have responded satisfactorily to most of my criticisms. I therefore recommend publication of the paper in BMJ open. However, I still think that the notion of anxiety should not be used in this article, especially in the keywords, as the authors did not measure this psychological variable with appropriate tools.

**Reviewer**	**3**
**Name**	**Eraso, Yolanda**
**Affiliation**	**London Metropolitan University, School of Social Sciences and Professions**
**Date**	**15-Sep-2024**
**COI**	**No competing interests**

Dear Colleagues,

Many thanks for the opportunity to review this revised manuscript.

My comments in relation to the earlier draft focused on specific areas of the Introduction and Discussion and I am satisfied that, with their additional comments, the authors have now addressed these concerns.
